# Success in the Dental Profession. No. 3

**Published:** 1862-08

**Authors:** C. C. Dills


### SUCCESS IN THE DENTAL PROFESSION.

BY C. C. DILLS, D. D. S.

### NO. 3: SYMPATHY; TENDERNESS; PATIENCE.

"He who will not give
Some portion of his ease, his blood, his wealth,
For another's good, is a poor frozen churl."—SHAKSPEARE.

I FULLY anticipate my inadequacy to write of sympathy, tenderness and patience; I would have some nurse of a thousand wounded soldiers do it; some lady,—a wife,—delicate and feeble—crowning a life's labor of love, in some crowded Shiloh hospital, by tender devotion to her dying husband, and soothing care to his dying comrades, who fell like *he* fell; or some venerable retired physician, whose head silvered o'er, and heart grown warm, reflects on the suffering he has seen, and sighs that there is so much, and that he has not been more sympathizing, more tender and patient; or still more to the point, some venerable, old dentist, whose kind heart never failed to appreciate the extent that sympathy, tenderness and patience could alleviate suffering in his profession.

"So many great,
Illustrious spirits have conversed with woe;
Have in her school been taught,—
How beautifully and poignantly could they write of these things."

But I must write, and can only trust the appeal of suffering, as it comes to one afar off, for inspiration.

It may be suggested here, that I am rather writing of philosophy than success, in the dental profession. But I imagine I am not going far astray, in advocating the good, even as a means to the genuinely profitable—so closely are they connected—aye, so certainly are our desert and success co-equal, throughout. It must be so, else has God not well enough protected the good. And with the case in hand,—else whence the expression, "I will get my dentistry of Dr. A.—he is so sympathizing, so tender, and patient! Dr. B. is so rough and unfeeling!—I shall never employ him again."

What dentist is not familiar with the fact, that as often as his work is spoken of, so also are his manners and sensibilities; and that with many the latter considerations seem paramount? Besides, there is not the same excuse, usually, for the deficient manner that there is for the imperfect work. A dentist with a good reputation for his work may almost as well as not add that for his manners and sensibilities.

Again, how beautiful are the workings of sympathy, aside from its virtue! Last night my little nephew, Ben, five years old, awoke from a sound sleep in a perfect paroxysm of pain from toothache. Did I not know that not possibly could I produce quiet and rest here, by any local application I could make? This, then, was a secondary consideration. To take him to my arms,—to caress him,—to suppress nervousness with pressure, and draw the pain from his face to mine by contact and sympathy;—these were my first considerations, and most potent.

Is this far fetched? By no means! Children are many times our patients, and as many times men are women of as delicate mould.

Sympathy and tenderness predominate in the young over the old practitioner; and, without claiming more for myself than this advantage of youth, I am fully satisfied I many times gain a profitable case, over competitors, through tenderness. I know of a town, with two dental practitioners, where it frequently occurs that a person goes to one of them to have his mouth prepared for artificial teeth, and to the other to have them inserted. I instance the case to the discredit of the latter—for with *tenderness*, he would avoid the danger of having his patients fall first in the hands of his competitor.

To instance a case verified by the observation of all, and from which the rule may be inferred, let me suppose a lady, distracted with pain, and trembling with fear, awaiting the dentist's appearance, to be relieved of obnoxious teeth. I have said the patient was distracted with pain—her mind "drawn apart, drawn in different directions, *diverted from its object.*" She is approached by an unfeeling, uncultivated dentist, to whom, as the proper person to hear it, she addresses a story most interesting to her, and well calculated, she thinks, to elicit sympathy for her. But the dentist (?) anticipates it—he has heard many such before, and is not now disposed to hear a repetition. He displays his forceps and vulgar impatience—she becomes more fearful, he becomes more unreasonable; she more distracted,—her powers more scattered, till now her strongest efforts at submission are futile; and in utter despair, she abandons all hope of success, censuring herself, perhaps, for a failure brought on by the *dentist* alone. For what dentist so ungenerous, to say the least of it, as not to grant a *necessity* attending the indecision of his patient *many times;* and so short-sighted as not to see that in such cases his only hope for success lies in patient waiting and gentle influences? Or what dentist so inconsiderate as to add to his own inability to submit to the excavation or extraction of a tooth—a peremptory refusal for the operator to proceed at all—and yet go straightway and forget what manner of man he is, by unflinchingly insisting that another shall submit to a like operation forthwith.

But to return. Instead of the uncultivated or unfeeling dentist, our patient is approached by one cultivated and kind. He, too, can anticipate her story, but he does not wish to,—or rather, he can not afford to; he makes haste slowly; he anticipates her *case* as well, and extends to her the sympathy it requires.

"Companionship in woe doth woe assuage."

Very rapidly does patient sympathy win her, and enable her to concentrate her powers wholly on the operation, which is *successful.* And thus often is success.

PIQUA, Ohio, *July*, 1862.

